# Supplementary material for: Comparison of cable bacteria genera reveals details of their conduction machinery
Source: EMBO Rep. 2025 Feb 17;26(7):1749–67. doi: 10.1038/s44319-025-00387-8 (PMC11976967; doi:10.1038/s44319-025-00387-8)
Supplement: Supplementary file 1 — Appendix [file 44319_2025_387_MOESM1_ESM.pdf]

Appendix for

Comparison of cable bacteria genera reveals details of their conduction machinery

Leonid Digel[1,2,3], Mads L. Justesen[1,3]#, Nikoline S. Madsen[1,3]#, Nico Fransaert[4], Koen Wouters[1,4,5], Robin Bonné[1,2], Lea E. Plum-Jensen[1,2], Ian P. G. Marshall[1,2], Pia B. Jensen[1,5], Louison Nicolas-Asselineau[1,6], Taner Drace[3,7], Andreas Bøggild[5,7], John L. Hansen[5,8], Andreas Schramm[1,2], Espen D. Bøjesen[5,9], Lars Peter Nielsen[1,2], Jean V. Manca[4], and Thomas Boesen[1,3,5,7]*.

Affiliations

[1]Center for Electromicrobiology, Aarhus University; 8000 Aarhus, Denmark.
[2]Department of Biology, Aarhus University; 8000 Aarhus, Denmark.
[3]Department of Molecular Biology and Genetics, Aarhus University; 8000 Aarhus, Denmark.
[4]X-LAB, UHasselt, 3500 Hasselt, Belgium.
[5]Interdisciplinary Nanoscience Center (iNANO), Aarhus University, 8000 Aarhus, Denmark
[6]Max Planck Institute for Marine Microbiology, 28359 Bremen, Germany
[7]EMBION - The Danish National Cryo-EM Facility – Aarhus Node, Aarhus University; 8000 Aarhus, Denmark.
[8]Department of Physics and Astronomy, Aarhus University; 8000 Aarhus, Denmark.
[9]Aarhus University Centre for Integrated Materials Research, Aarhus University, 8000 Aarhus, Denmark.
# These authors contributed equally.
* Corresponding author: Thomas Boesen, thb@inano.au.dk

List of Appendix Figures and Tables

Appendix Tables

Appendix Table S1	Page 3
Appendix Table S2	Page 4

Appendix Figures

Appendix Figure S1	Page 5
Appendix Figure S2	Page 6
Appendix Figure S3	Page 7
Appendix Figure S4	Pages 8-10
Appendix Figure S5	Page 11
Appendix Figure S6	Page 12
Appendix Figure S7	Page 13
Appendix Figure S8	Page 14
Appendix Figure S9	Page 15
Appendix Figure S10	Page 16

Appendix Table S1. Filamentous bacteria for which conductivity was non-detectable (< 1 pA, at 1 V on interdigitated electrodes)

	Source/Reference	Species/Genus	Conductivity (S/cm)
**Pure cultures**	Italian National Research Council, Rome University	*DYN-VER9-ISO2* related to *Kouleothrix. aurantiaca*	$< 10^{-7}$
	Italian National Research Council, Rome University	*Ca.* Meganema perideroedes	$< 10^{-7}$
	Italian National Research Council, Rome University	Unknown species within Cytophaga-Flavobacterium-Bacteroides group	$< 10^{-7}$
	The Leibniz Institute DSMZ (5205)	*Thiothrix nivea*	$< 10^{-7}$
	The Leibniz Institute DSMZ (14523)	*Anaerolinea thermophila*	$< 10^{-7}$
	The Leibniz Institute DSMZ (16556)	*Leptolinea tardivitalis*	$< 10^{-7}$
	The Leibniz Institute DSMZ (23923)	*Pelolinea submarina*	$< 10^{-7}$
	The Leibniz Institute DSMZ (14018)	*Geothrix fermentans*	$< 10^{-7}$
	The Leibniz Institute DSMZ (21853)	*Caldiserica exile*	$< 10^{-7}$
	Kawaichi et al., (2)	*Ardenticatena maritima*	$< 10^{-7}$
	Chailakhyan et al., (3)	*Phormidium unicatum*	$< 10^{-7}$
	Pasteur Culture Collection for Cyanobacteria (PCC)	*Geitlerinema PCC 9228*	$< 10^{-7}$
**Field samples**	Ferry Harbor, Grenå (56.406431, 10.921926)	*Beggiatoa* sp.	$< 10^{-7}$
	Schleswig sediment (54.587389, 9.830481)	*Crenothrix* sp.	$< 10^{-7}$
	Aggersund (56.997181, 9.302312)	*Microcoleus* sp.	$< 10^{-7}$

Appendix Table S2. Inner membrane vesicle dimensions from different cryo-ET tilt series collected on Electronema aureum GS and Electrothrix communis RB. The sample numbers designate the tilt-series code in the raw data. IMAV – inner membrane-attached vesicle, CV – cytoplasmic vesicle. NA – not applicable. '?' designates measurements that were limited by the signal/noise ratio of the tilt-series.

Sample name	Vesicle count		Vesicle D (mean), nm		% of IMAV
	IMAV	CV	IMAV	CV	
Electronema aureum GS 3	8	24	64.7	67.8	25
Electronema aureum GS 13	17	20	69.1	71.0	45.9
Electronema aureum GS 17	28	1	66.4	NA	96.6
Electronema aureum GS 40	15	1	63.3	NA	93.8
Electronema aureum GS 41	12	2	62.9	NA	85.7
Electronema aureum GS 42	10	0	64.2	NA	100
Electrothrix communis RB 0	19	0	59.4	NA	100?
Electrothrix communis RB 1	22	0	56.3	NA	100?
Electrothrix communis RB 2	28	0	57.4	NA	100?

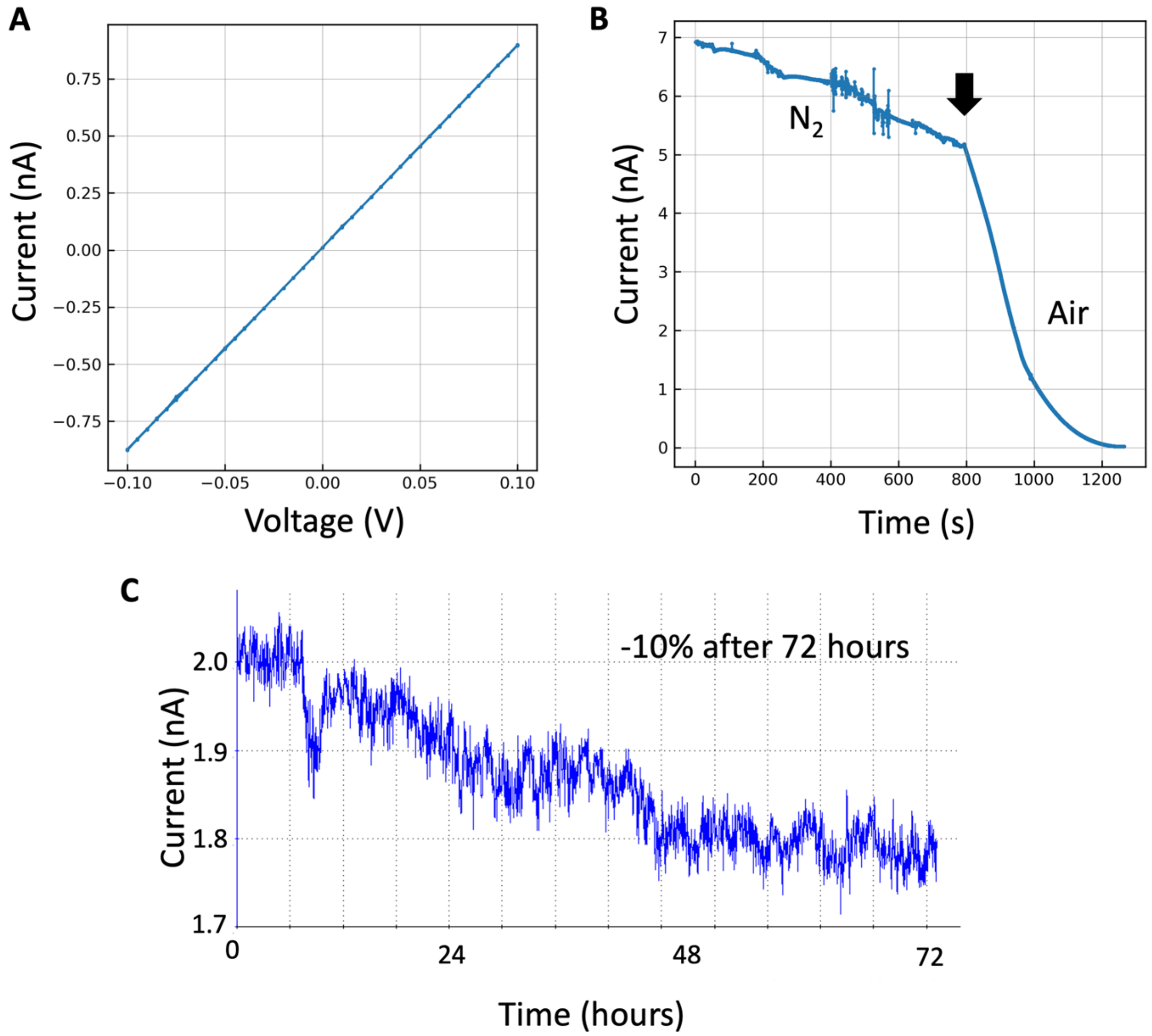

Appendix Figure S1. Conductivity characteristics of cable bacteria. (**A)** The highly linear current response to the voltage; (**B)** The slow degradation in a N_2 atmosphere, and fast degradation in air (black arrow shows when air was introduced); (**C)** The stability of electrical conductivity in a vacuum at 100mV.

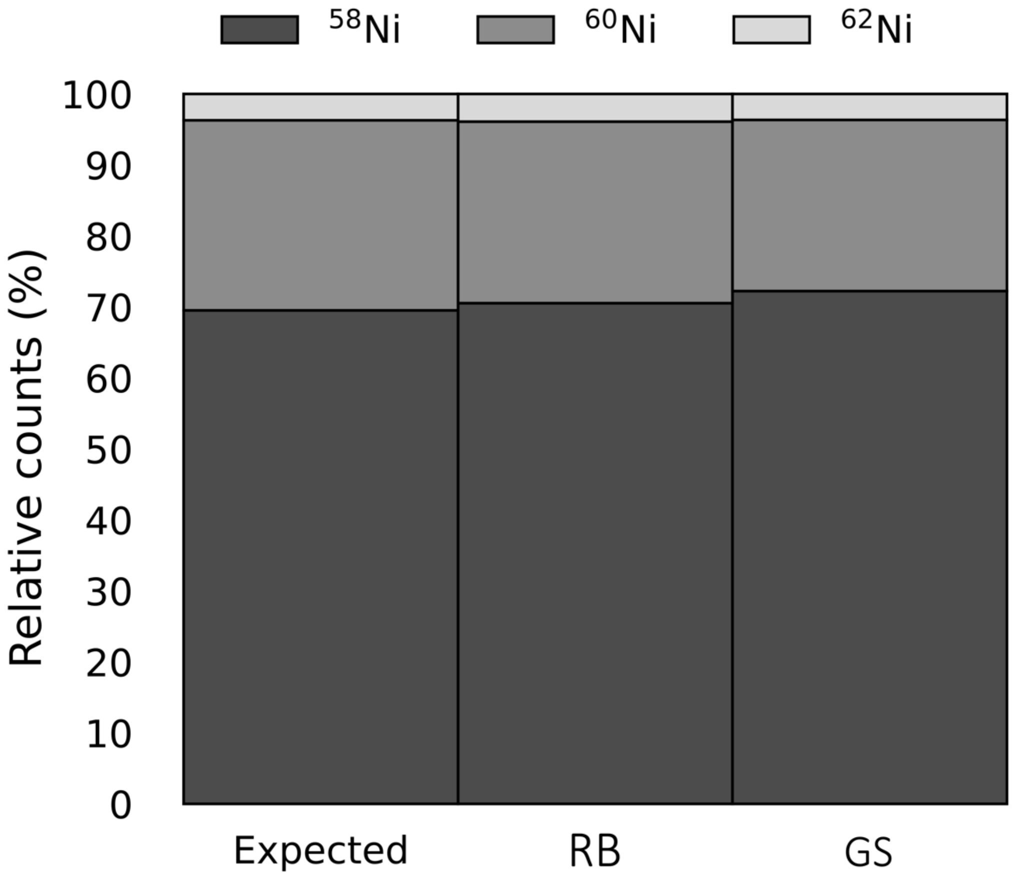

Appendix Figure S2. Isotope analysis of nickel for both strains, RB, and GS, demonstrates agreement between the observed and expected counts, indicating that nickel is correctly identified.

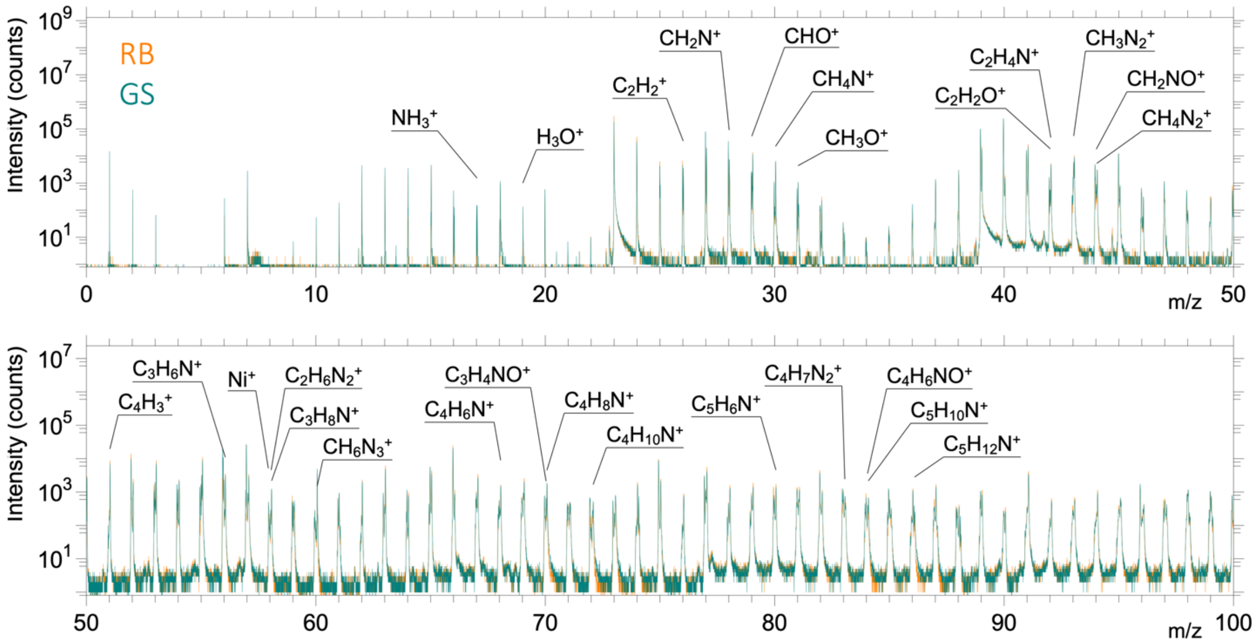

Appendix Figure S3: The ToF-SIMS spectra of intact RB and GS filaments exhibit similarities. The annotated fragments have been associated to protein and polysaccharide layers in previous studies (6, 7).

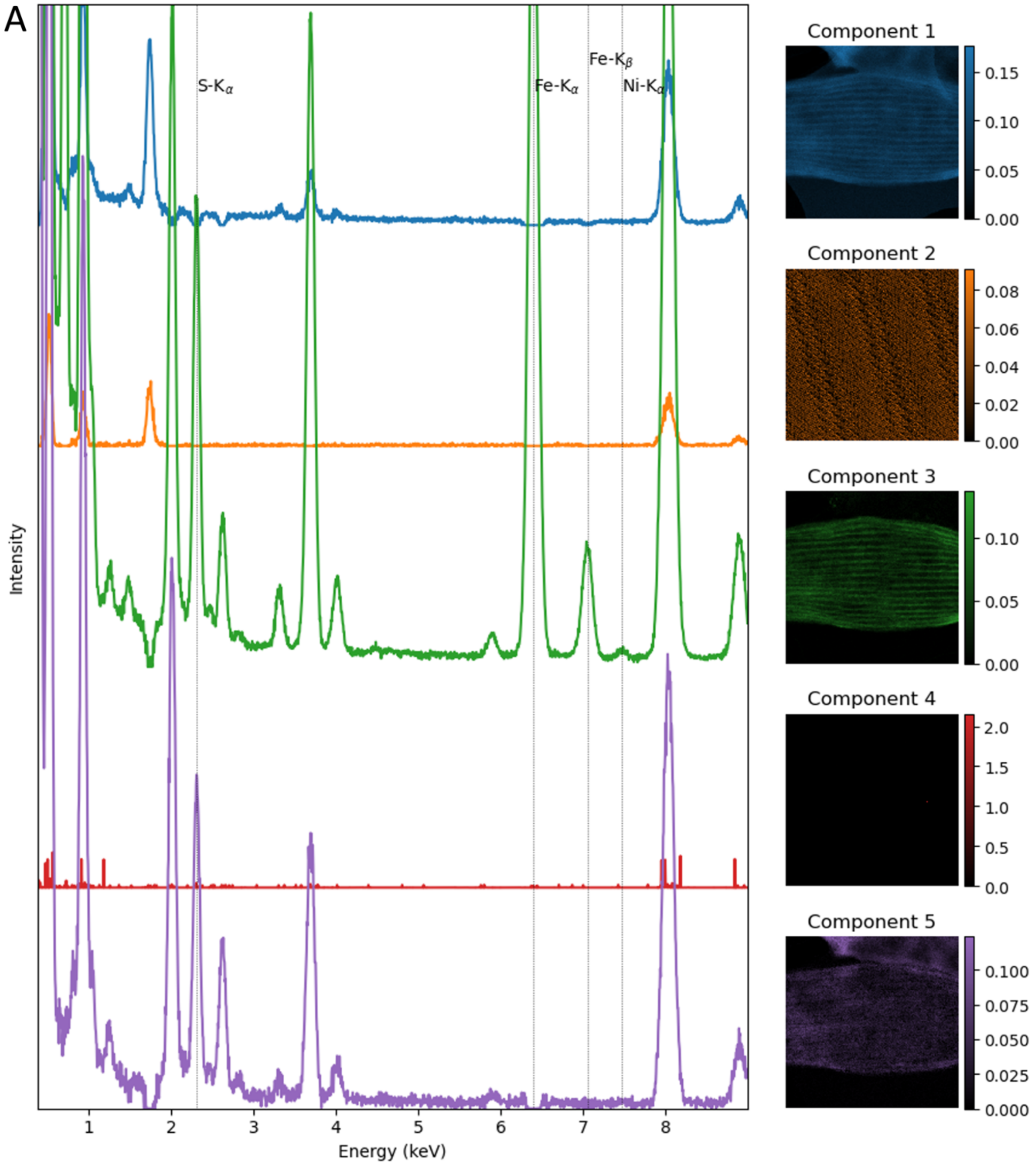

A
S-K$_\alpha$
Fe-K$_\alpha$
Fe-K$_\beta$
Ni-K$_\alpha$
Intensity
Energy (keV)
1
2
3
4
5
6
7
8
Component 1
0.15
0.10
0.05
0.00
Component 2
0.08
0.06
0.04
0.02
0.00
Component 3
0.10
0.05
0.00
Component 4
2.0
1.5
1.0
0.5
0.0
Component 5
0.100
0.075
0.050
0.025
0.000

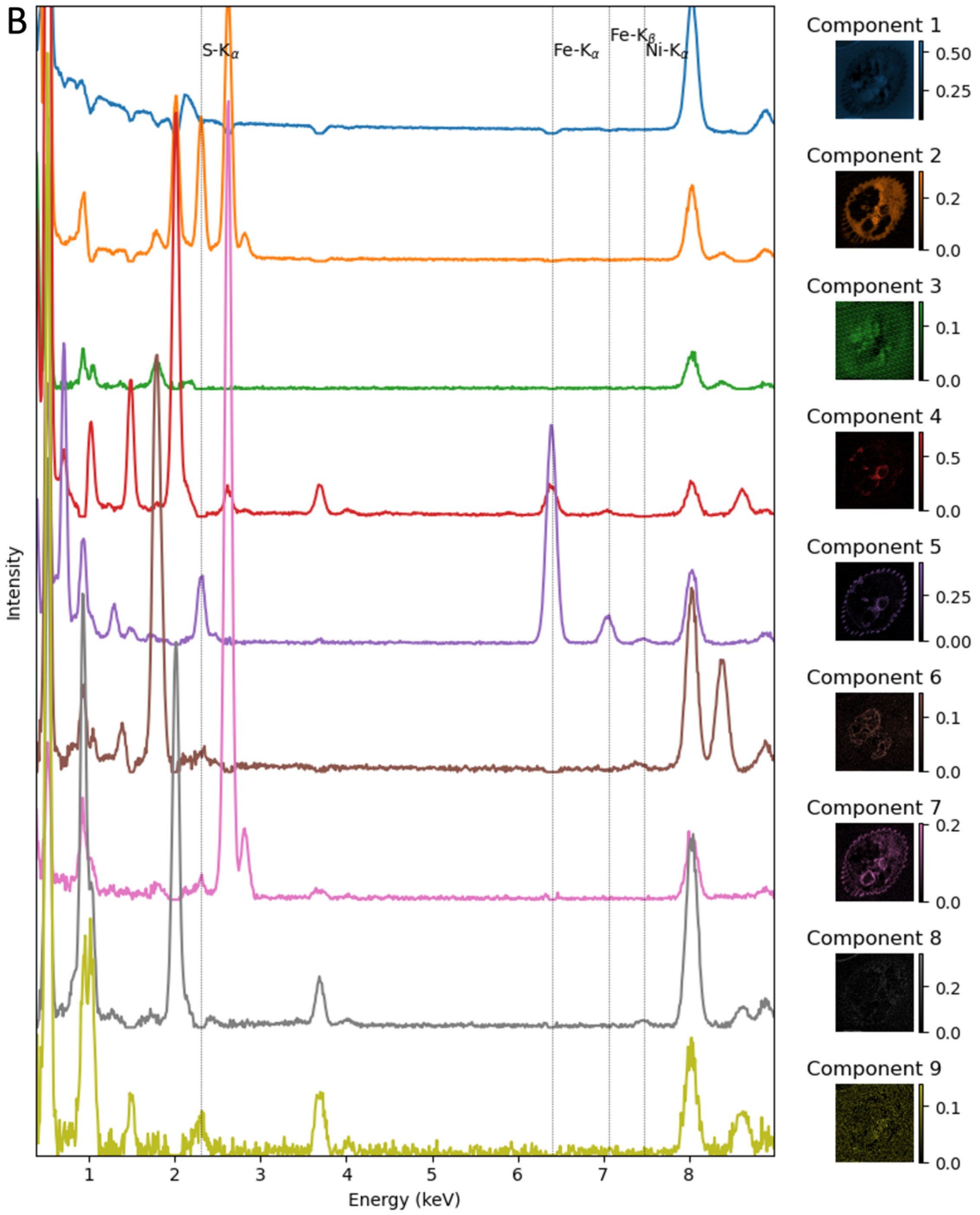
B
S-K$_\alpha$
Fe-K$_\alpha$
Fe-K$_\beta$
Ni-K$_\alpha$
Intensity
Energy (keV)
1
2
3
4
5
6
7
8
Component 1
0.50
0.25
Component 2
0.2
0.0
Component 3
0.1
0.0
Component 4
0.5
0.0
Component 5
0.25
0.00
Component 6
0.1
0.0
Component 7
0.2
0.0
Component 8
0.2
0.0
Component 9
0.1
0.0

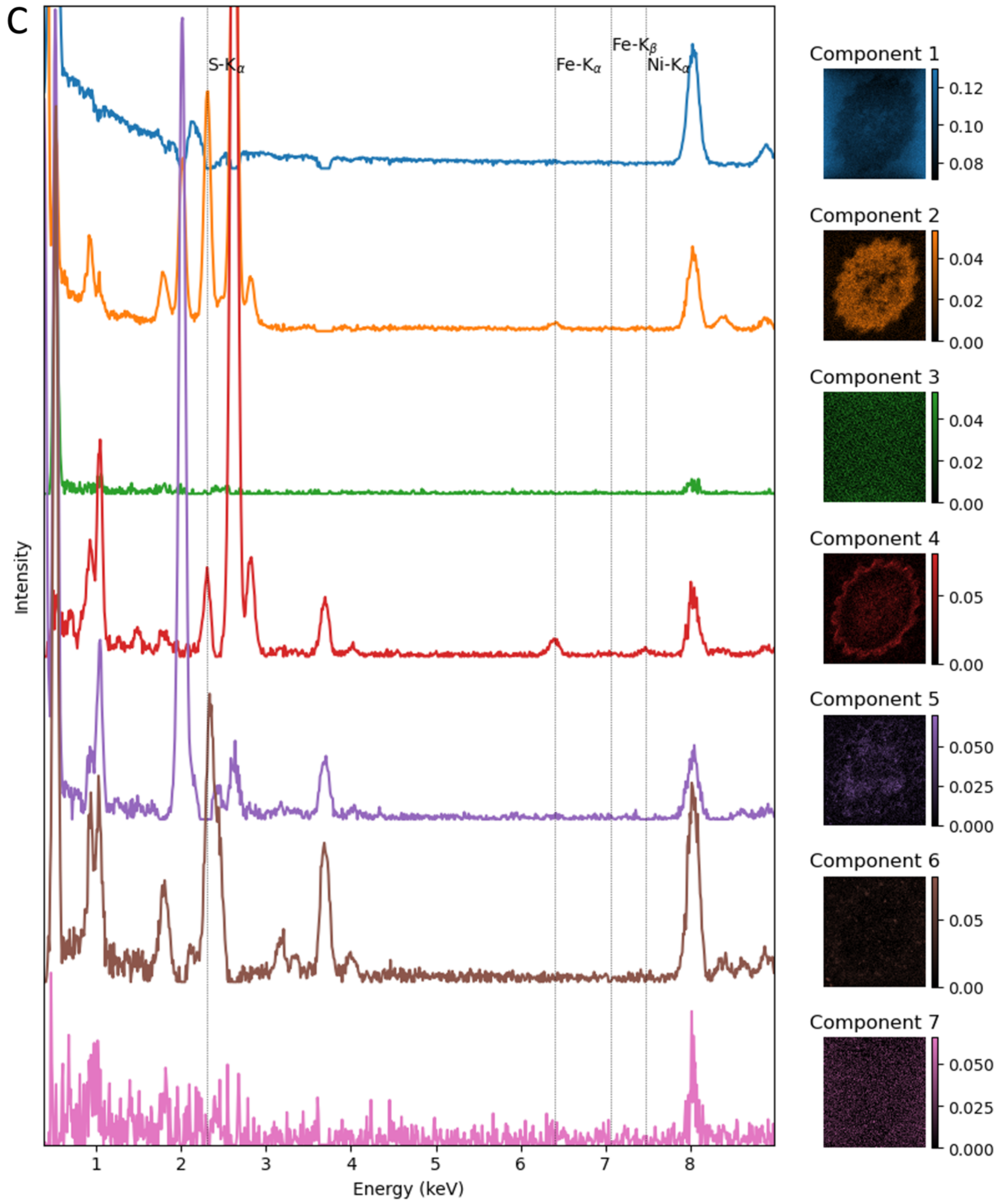

Appendix Figure S4. Non-negative matrix factorization decomposition of STEM-EDX spectrum images. (**A**) an intact GS cell, (**B**) a GS cross-section, and (**C**) an RB cross-section. The extracted sum spectra for each component and relative intensities of components in the sample are shown on the left and right, respectively.

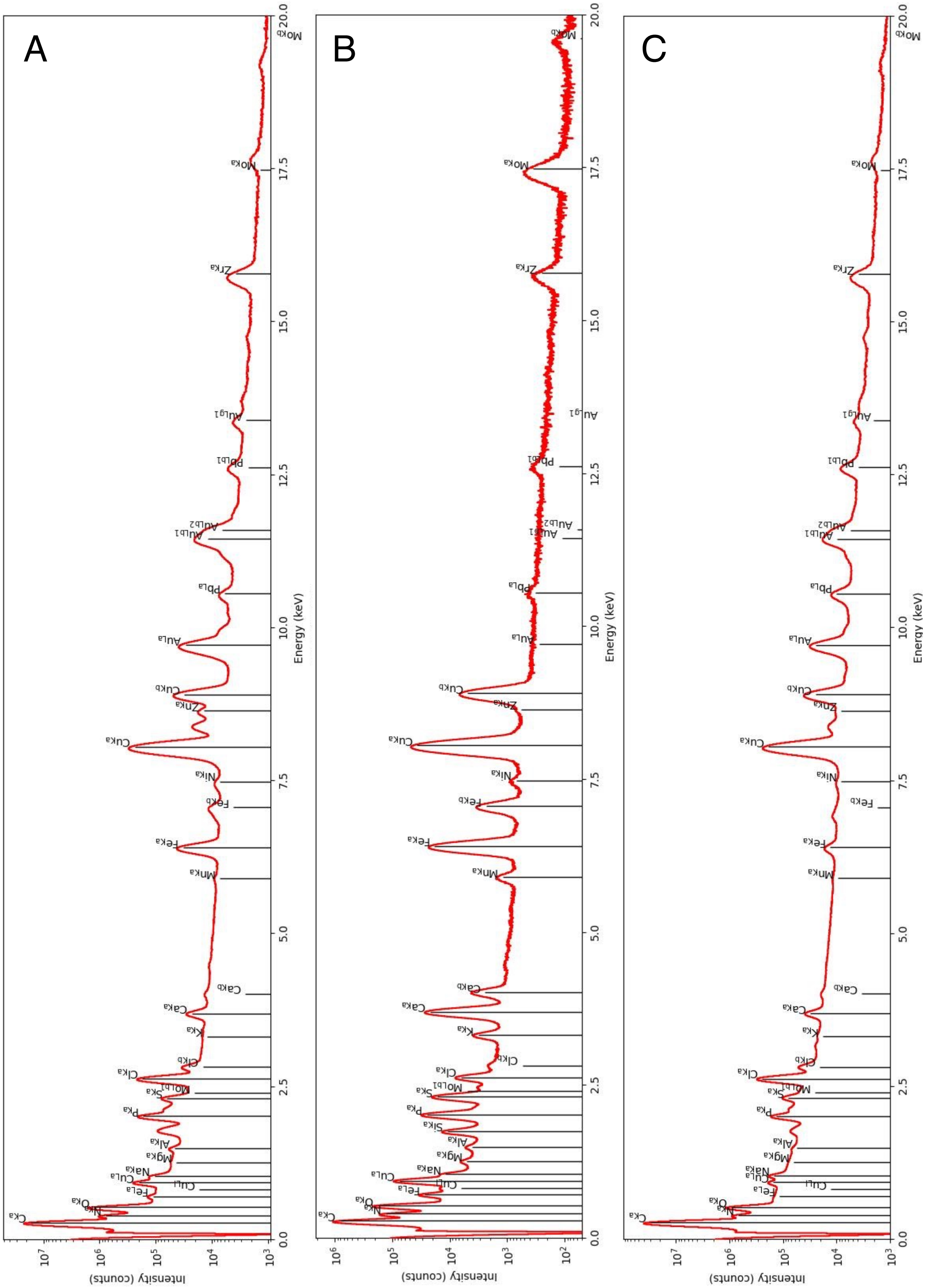

Appendix Figure S5. (A) Summed spectrum of intact GS cell. (**B)** Summed spectrum of GS cross-section. (**C)** Summed spectrum of RB cross-section.

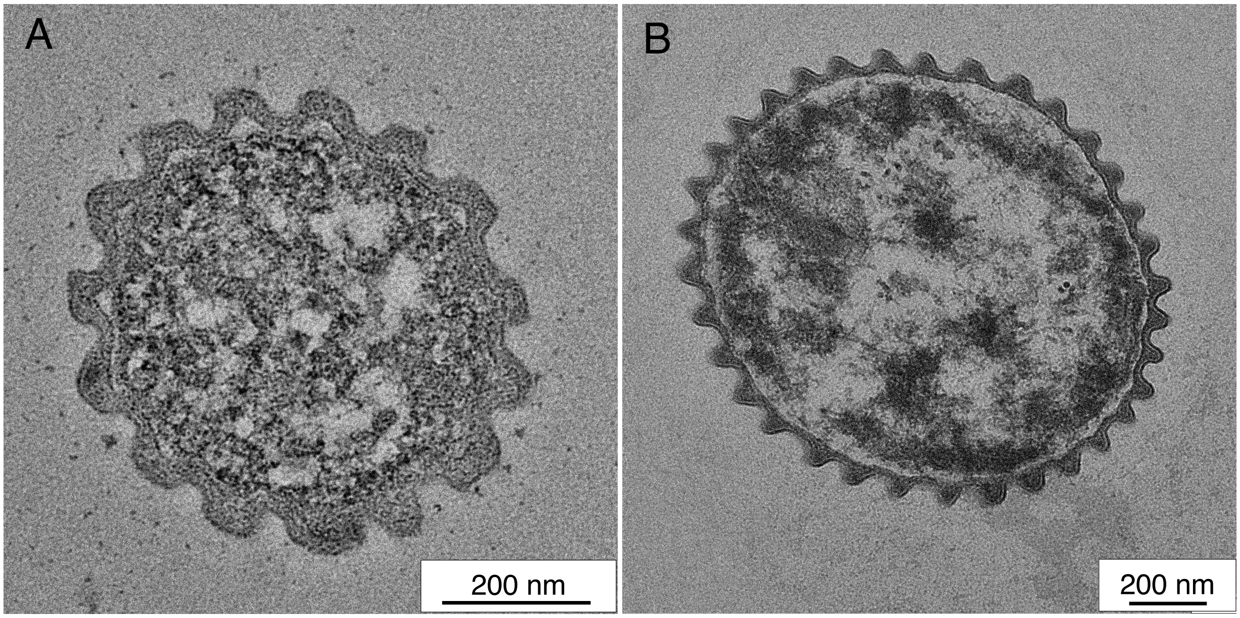

Appendix Figure S6. TEM of plastic-embedded cross sections from cable bacteria filaments. (A) RB cross-section showing mostly rectangular PCFs and (**B)** GS cross-section with consistent rounded shape of the PCFs. Note the difference in scale bars. The images are re-used from Figure 1A and presented here at a similar visual scale to demonstrate the difference in PCF morphology between different cable bacteria.

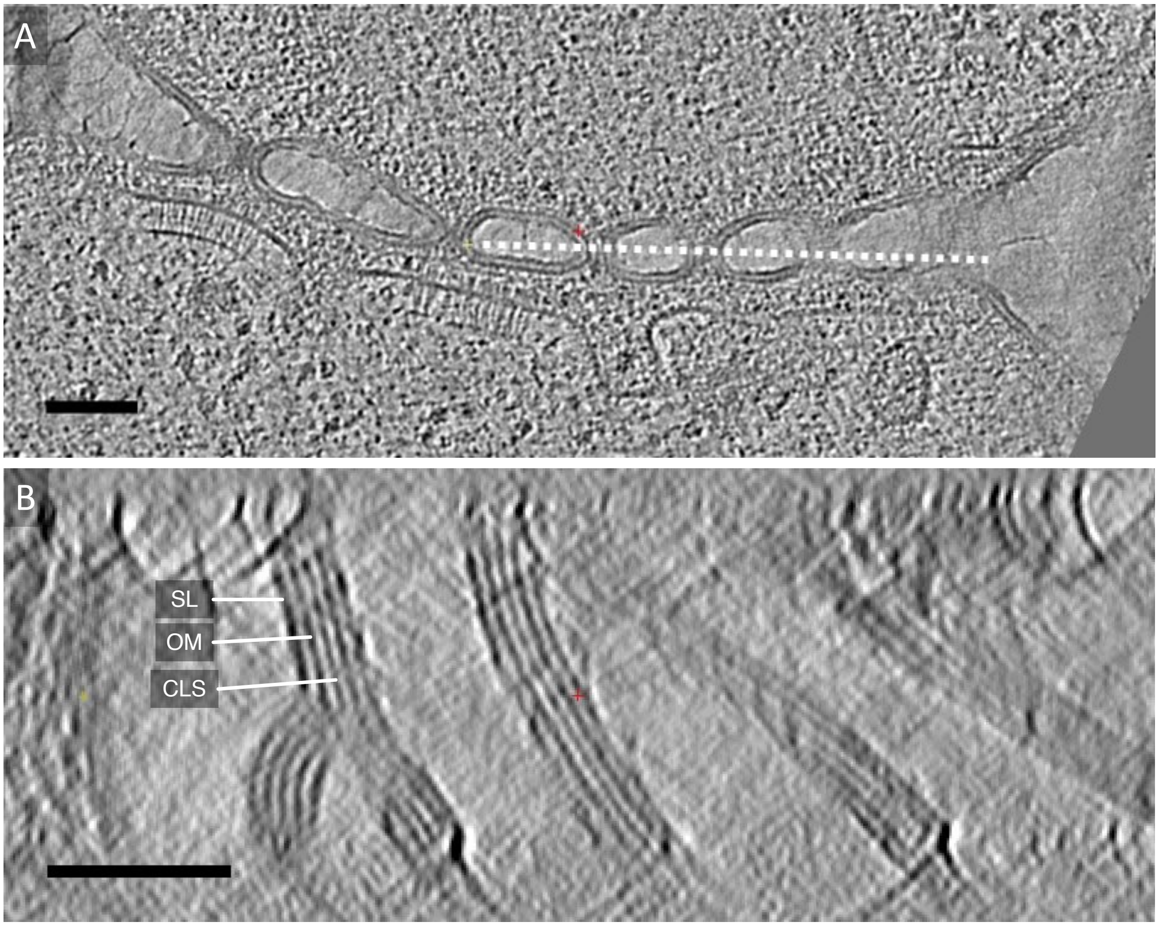

Appendix Figure S7. The ultrastructural organization of the cable bacteria cell-cell junction. (A) Tomographic slice of a cable bacteria cell-cell junction showing a longitudinal section of the junction lamella. (**B)** Tomographic slice through the junction lamella in the position shown by the white dashed line in A showing branching and the high-resolution details of the CLS. The top of the image is facing the center of the junction. CLS – core lamella sheet, SL – surface layer, OM – outer membrane. Scale bars: 100 nm.

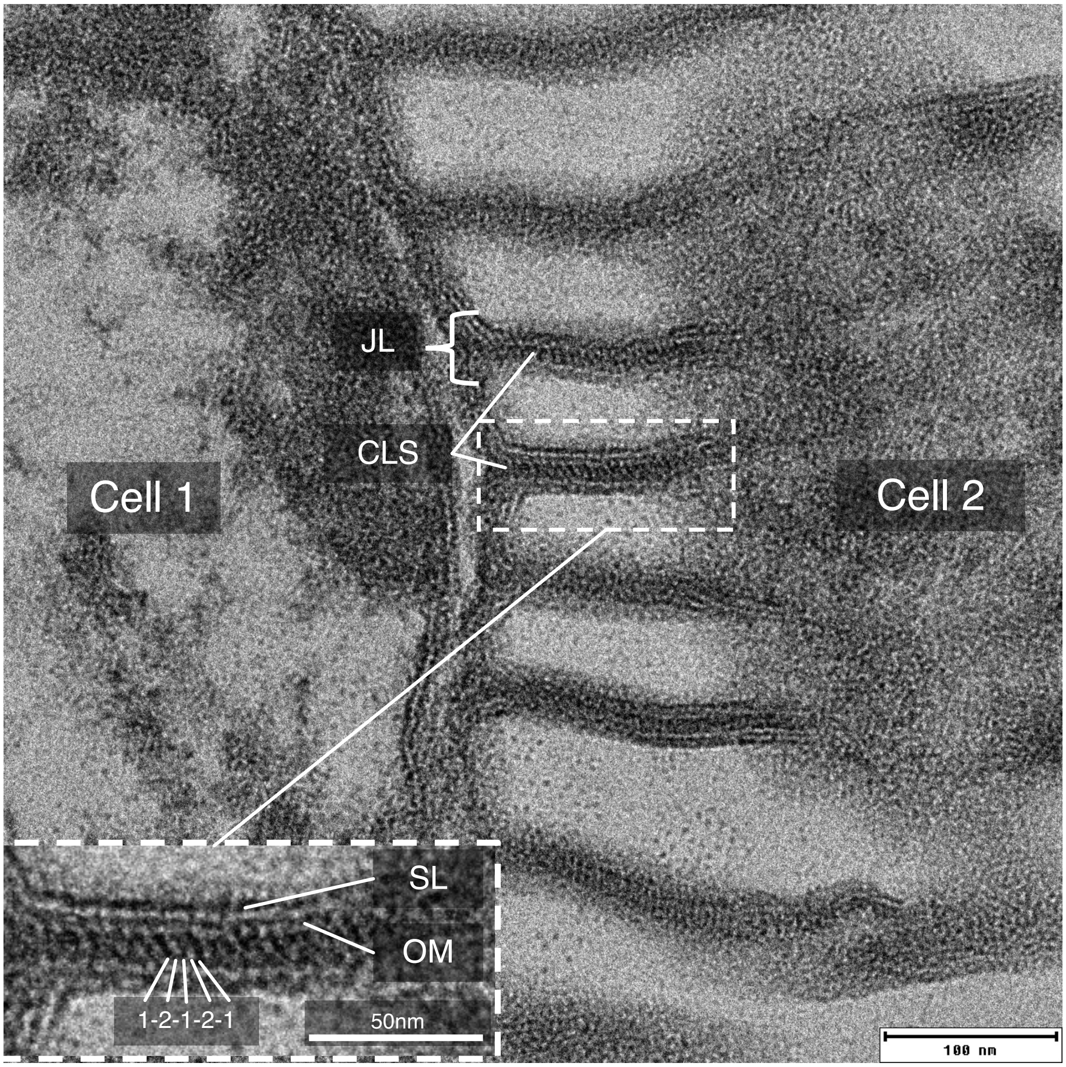

Appendix Figure S8. Longitudinal section of a cell-cell junction of a GS cable bacterium embedded in plastic. Dashed rectangle outlines the region zoomed-in in the figure insert. The insert presents the architecture of the junction lamella, in which a repeated pattern can be seen. JL- junction lamella, CLS – core lamella sheet, SL – surface layer, OM – outer membrane, 1 and 2 represent the repeats in the CLS structure.

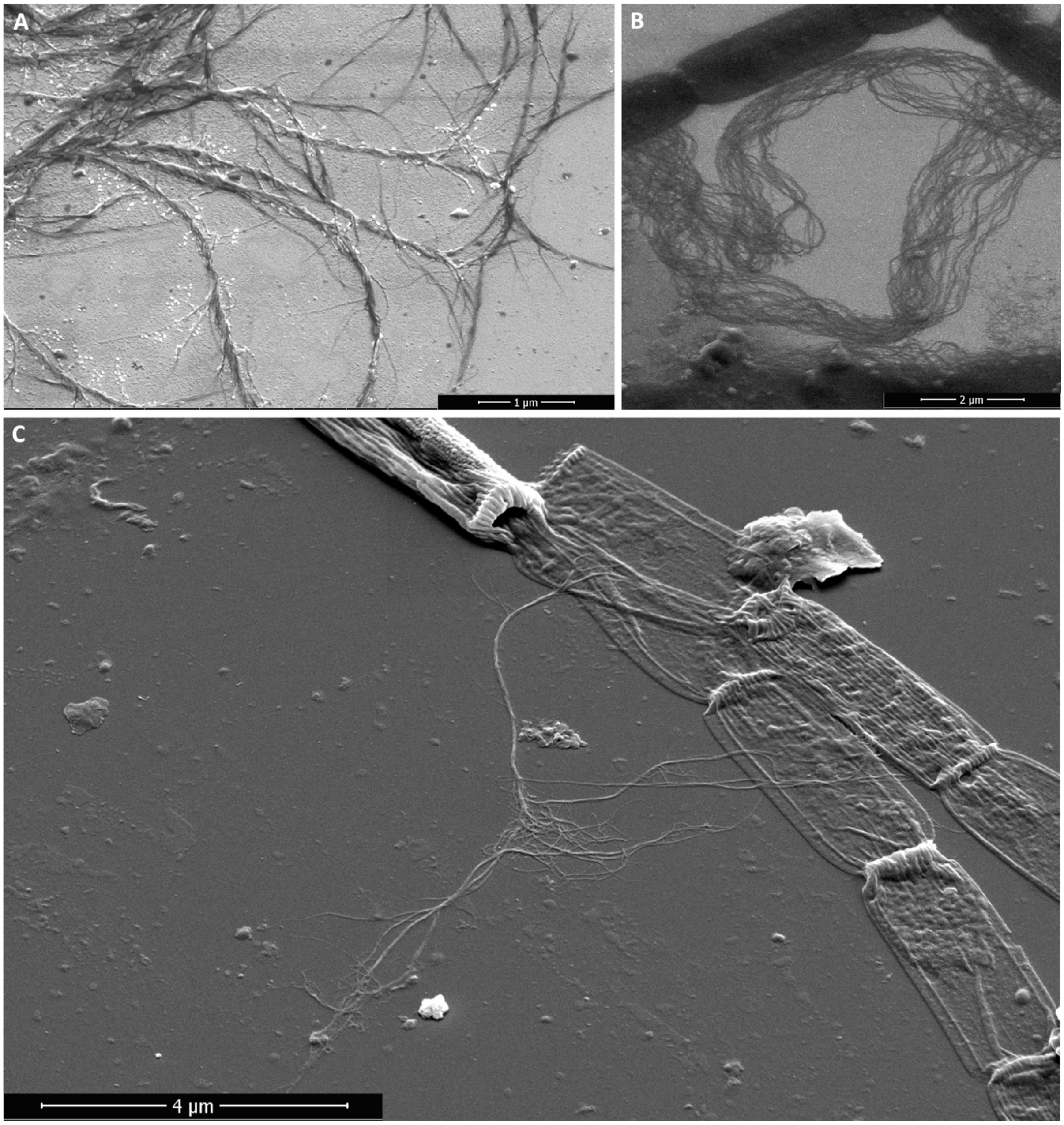

Appendix Figure S9. SEM images of PCF strand components from different cable bacteria filaments. (A, B) GS released PCF strand components after harsh mechanical treatment (**C)** Marine cable bacteria from Hou beach released PCF strand components after 100min incubation in 1% SDS.

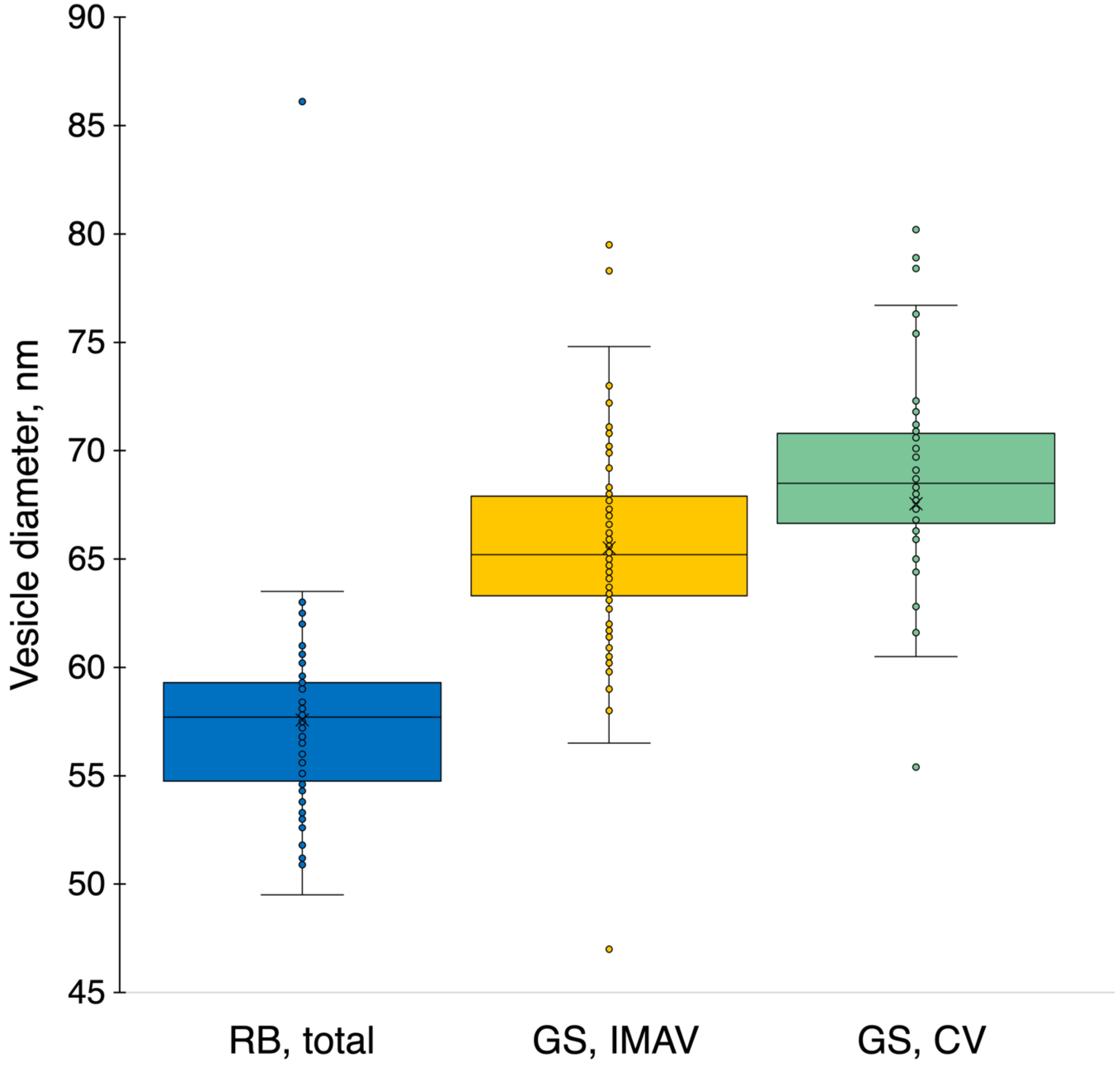

Appendix Figure S10. Total measurements of the diameters of Inner Membrane-attached Vesicles (IMAVs) and Cytoplasmic Vesicles (CVs) from RB and GS cable bacteria compiled together for comparison. RB total n=69, GS IMAV n=90, GS CV n=48. The bold line denotes the median (center), the lower and upper bounds of box the 25th and 75th percentiles and the whiskers extend to the minima and maxima values no larger than 1.5× inter-quartile range. Values outside of the boundary of the whiskers are outliers. Each point is a biological replicate.
