# Supplementary material for: Comparison of cable bacteria genera reveals details of their conduction machinery
Source: EMBO Rep. 2025 Feb 17;26(7):1749–67. doi: 10.1038/s44319-025-00387-8 (PMC11976967; doi:10.1038/s44319-025-00387-8)
Supplement: Supplementary file 6 — Expanded View Figures [file 44319_2025_387_MOESM6_ESM.pdf]

Expanded View Figures

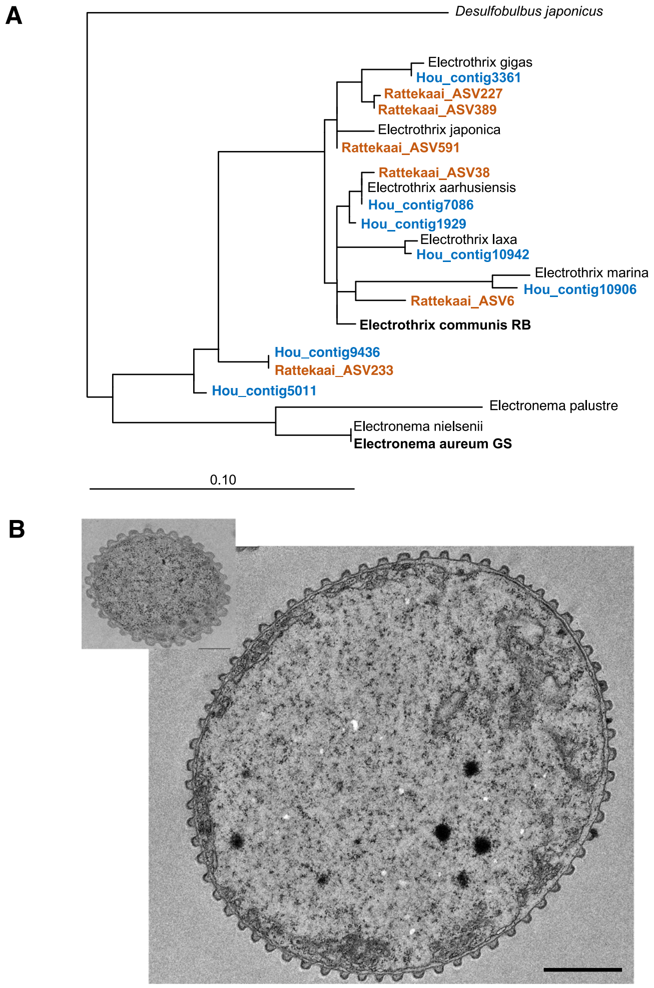

Figure EV1. Cable bacteria diversity and morphology in the additional samples used for conductivity measurements and based on 16S rRNA gene sequencing.

(**A**) Blue, sequences extracted from metagenome sequencing of Hou beach. Orange, data from amplicon sequencing of Rattekaai sediment. Black, reference sequences, with single-strain cable bacteria cultures in bold. A maximum likelihood tree was calculated in ARB using near-complete 16S rRNA gene sequences, and partial (ASV) sequences were added using the maximum likelihood tool without changing the tree topology. The phylogenetic analysis showed that both marine sites exclusively contained cable bacteria of the genus Electrothrix and indicates at least 5 different Electrothrix species in the Rattekaai samples, and at least 7 different Electrothrix species in the Hou beach samples. Scale bar, 0.1 base changes per site. (**B**) Representative TEM images of plastic-embedded cross sections of cable bacteria cells sampled from Hou beach (top) and Rattekaai sediment (bottom). Scale bar: 1 µm.

© The Author(s)

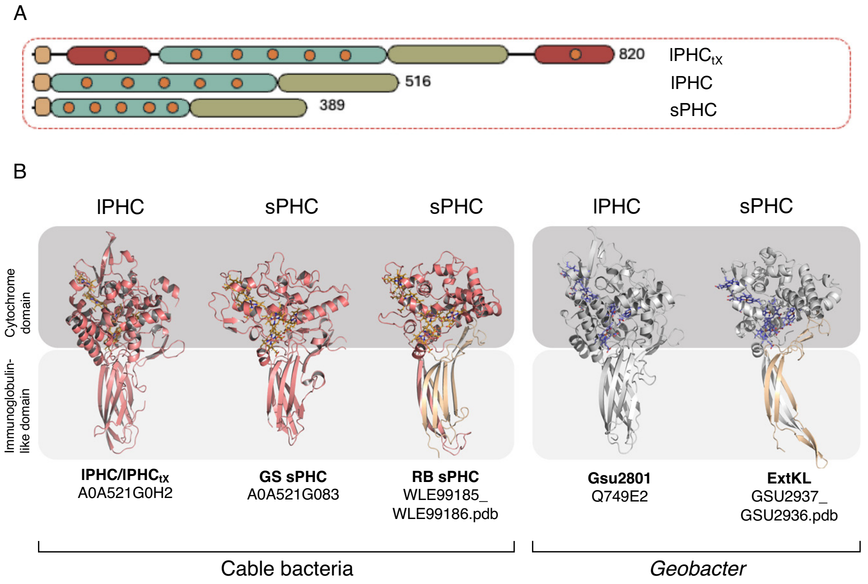

Figure EV2. The conserved pentaheme cytochrome (PHC) family.

(**A**) Schematic of the domain structure of PHC family members from GS cable bacteria. Orange circles—hemes, red—truncated hemoglobin domain, green—immunoglobulin-like domain, cyan—PHC domain, yellow square—signal peptide. (**B**) The structural models of the proposed pentaheme cytochrome family, that is conserved in both cable bacteria and *Geobacter*. Folding of the downstream genes (wheat) for RB sPHC and ExtKL results in a predicted structure with the larger immunoglobulin-like domain and similar overall fold to the other PHCs.
